# Single-cell RNA sequencing of murine hearts for studying the development of the cardiac conduction system

**DOI:** 10.1038/s41597-023-02333-6

**Published:** 2023-09-04

**Authors:** Huiying Ren, Xiaolin Zhou, Jun Yang, Kun Kou, Tangting Chen, Zhaoli Pu, Kejun Ye, Xuehui Fan, Dan Zhang, Xinjiang Kang, Zhongcai Fan, Ming Lei, Tianyi Sun, Xiaoqiu Tan, Xianhong Ou

**Affiliations:** 1 Key Laboratory of Medical Electrophysiology of Ministry of Education and Medical Electrophysiological Key Laboratory of Sichuan Province, Institute of Cardiovascular Research, Southwest Medical University, Luzhou, 646000 China; 2 Department of Cardiology, The Affiliated Hospital of Southwest Medical University, Luzhou, 646000 China; 3 Department of Pharmacology, University of Oxford, Oxford, OX1 3QT United Kingdom; 4 Department of Physiology, School of Basic Medical Sciences, Southwest Medical University, Luzhou, 646000 China

**Keywords:** Genetics research, Gene ontology

## Abstract

The development of the cardiac conduction system (CCS) is essential for correct heart function. However, critical details on the cell types populating the CCS in the mammalian heart during the development remain to be resolved. Using single-cell RNA sequencing, we generated a large dataset of transcriptomes of ~0.5 million individual cells isolated from murine hearts at six successive developmental corresponding to the early, middle and late stages of heart development. The dataset provides a powerful library for studying the development of the heart’s CCS and other cardiac components. Our initial analysis identified distinct cell types between 20 to 26 cell types across different stages, of which ten are involved in forming the CCS. Our dataset allows researchers to reuse the datasets for data mining and a wide range of analyses. Collectively, our data add valuable transcriptomic resources for further study of cardiac development, such as gene expression, transcriptional regulation and functional gene activity in developing hearts, particularly the CCS.

## Background & Summary

The cardiac conduction system (CCS) is a specialized tissue that coordinates the rhythmic contractions of heart muscle by controlling the generation and propagation of the causative electrical impulse. Failure to correctly pattern and develop the CCS components leads to several cardiac diseases^1^. The CCS includes the sinoatrial node (SAN), atrioventricular node (AVN), His bundle, bundle branches and Purkinje fibre (PF) network^2^. Each of these components is highly specialized but contains heterogeneous cell types with distinct electrophysiological properties^2–4^. Our understanding about when and how these specialized cell types arise to form distinct CCS components remains limited^2^. In murine hearts, the electrical activity can be detected as early as E8^5^; yet the whole CCS is not completely formed until E16.5^2^. The SAN develops first in the CCS from within the sinus venosus myocardium of the heart tube. It can be recognized morphologically from E11.5 onwards in mice in the right sinus horn at the junction with the atrium^6^. The SAN and the atrioventricular conduction system (AVCS) develop simultaneously in the E11 to E12 mouse embryo heart^6^. While the establishment of the VCS occurs at mid-to-late fetal stages from E12.5 to 16.5, the PF network is completed perinatally^7^, the cellular origin of the various components of the CCS, particularly the VCS, is still in debate.

The recent emerging RNA sequencing (RNA-seq) technology has allowed for the fast quantification and characterization of transcriptomes. Integrating high-throughput data with computational and statistical methods provides a toolbox to study the molecular signatures of tissues^8^. The recent development of transcriptomic technologies, particularly the single-cell RNA-seq (scRNA-seq), has significantly improved our capability for studying cell populations such as revealing and characterizing novel cell types. By sequencing the genomes of a large number of single cells from an individual ‘sample’, scRNA-seq can detect the cellular components present in complex tissues^9–11^, identify unknown or rare cell types, clarify the changes of gene expression in the process of differentiation or time and state changes, find out the genes that are differentially expressed in a specific type of cells under different conditions (such as dosing and disease groups), and explore changes in gene expression between cell types, incorporating spatial, regulatory, and/or protein information. scRNA-seq also identifies unknown or rare cell populations that could not be resolved using bulk RNA-seq.

Moreover, scRNA-seq may also be used for tracking cell lineage during differentiation, as movement between different cell types is associated with changes in gene expression^12^. Recent studies have applied scRNA-seq to study cardiogenesis, focusing on cell populations by using defined genes^13–17^. Given our incomplete understanding of CCS morphogenesis and maturation, it is necessary to establish a sophisticated approach enabling the analysis of organ-wide spatial gene expression profiles without biasing against cellular heterogeneity.

**Fig. 1:**
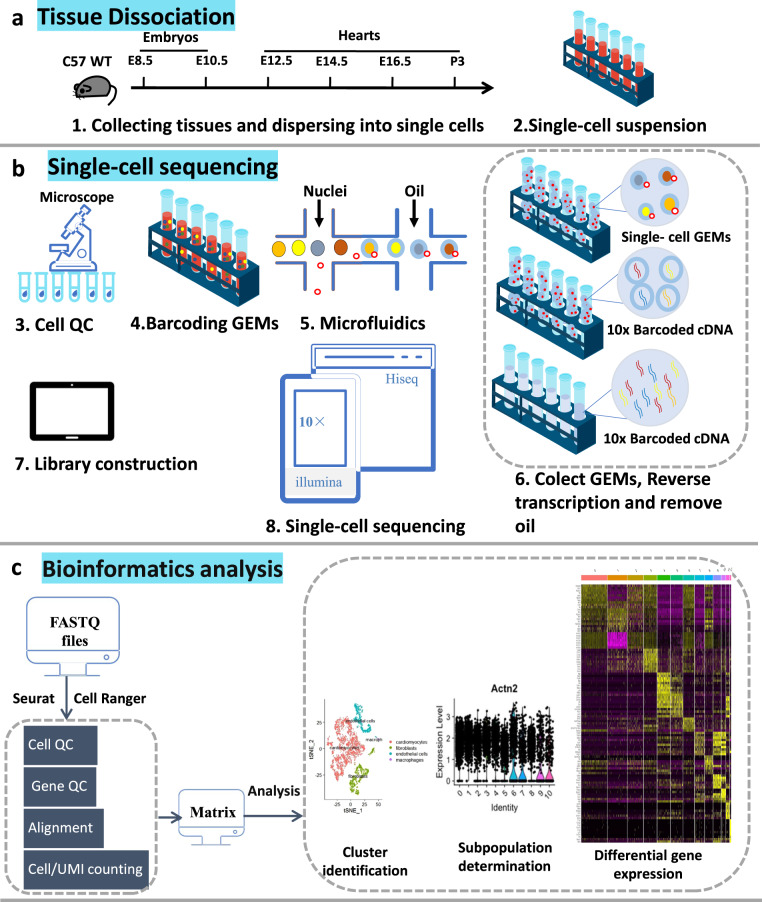
Schematic diagram of the 10 × single-cell RNA sequencing workflow. (**a**) Tissue dissociation. Mouse hearts of different periods were obtained in batches. Single-cell suspensions were prepared by enzymatic digestion. **(b)** Single-cell sequencing. The cell viability was detected to meet the experimental requirements. Then sequencing experiments were performed. (**c**) Bioinformatics analysis.

Figure 1 illustrates a schematic overview of the study design, from model generation, characterization, living heart slicing, high-throughput optical imaging, data processing and analysis. Using single-cell RNA sequencing, we generated a large dataset of transcriptomes of ~0.5 million individual cells isolated from murine hearts at six successive developmental stages corresponding to the early, middle and late heart development.

### The current dataset has the following features

Our dataset contains a large number of single-cell transcriptomes from six mid-to-late developmental stages by scRNAseq. We could discriminate rarer cardiac cell types, such as the CCS, through meaningful cardiomyocyte-focused quality control and utilizing a novel local and global structure-preserving dimensionality reduction technique. The dataset helps us understand the differentiation pattern of CCS in time and space. At the same time, the upstream and downstream targets of key transcription factors related to CCS development were deeply studied to clarify these transcription factors’ molecular characteristics and biological functions and provide new ideas for clinical diagnosis and treatment of arrhythmic diseases in the future.

## Methods

### Animals

Wild-type C57Bl/6 J mice at six developmental stages, including E8.5, E10.5, E12.5, E14.5, E16.5 and postnatal day 3 (P3) were used in this study, and suppied by Laboratory Animal Center of Southwest Medical University. After sacrificing the mice, the embryos (E8.5 and E10.5) and hearts (E12.5, E14.5, E16.5 and P3) were dissected. Single-cell suspensions were prepared as detailed below. The experimental animal ethics committee approved all animal experiments at Southwest Medical University, Sichuan (China) (No: 20160930).

### Preparation of cell suspensions for single-cell RNA sequencing analysis

We isolated and collected the single cells from embryos or hearts at six developmental stages, including E8.5, E10.5, E12.5, E14.5, E16.5 and postnatal P3 using the standard enzymatic method described previously^18^. E8.5 and E10.5 embryos were dissected from pregnant mice’s uterus and digested with collagenase II digestive solution (collagenase II: bovine serum albumin: DMEM/F12 = 0.01: 0.1: 10) after the cut-off in the head and limbs. The hearts from E12.5, E14.5, and E16.5 stages were dissected directly from the embryos. The operations were performed under a posture microscope: cut open the uterus, sequentially open the amniotic membrane, and remove the embryo. The embryo can flow out under the action of amniotic fluid and cut the umbilical cord. Then, the hearts of E12.5, E14.5, and E16.5 were removed from the embryonic for digestion. The specific digestion steps of each embryonic stage are similar. Hearts of P3 stage were dissected directly from the aortic root of the postnatal mice. After trimming excess connective tissue, thymus, lung tissue, and vascular tissue such as superior and inferior vena cava at the base of the heart, hearts were digested with collagenase II digestive solution.

During the single-cell suspension preparation, cell viability and concentration were detected by staining with 0.4% trypan blue. After primary quality control, cell viability was adjusted to the appropriate concentration for 10 × scRNA-seq. The diameter of cardiomyocytes in embryonic and postnatal mice ranged from 8 to 15 μm, meeting the standard requirements. The cell concentration can be controlled within 700–1200 cells/µL according to the concentration requirements. 8,000–16,000 cells were captured by the system in each sample (Table 1). The cell viability and agglomeration rate are shown in Table 2. The clustering rate is less than 5%, and the number of clustered cells can be seen under the microscope. There are basically no impurities and cell debris. It is considered that the cell quality is qualified and meets the sampling conditions for single-cell sequencing.

**Table 1:** Cellranger count in each sample.

Sample	Developmental stage	Cellranger count
E8_5_1_1	E8.5	5643
E8_5_2_2	E8.5	5380
E10_5_1	E10.5	14906
E10_5_2	E10.5	14703
E12_5_1_1	E12.5	5674
E12_5_2_2	E12.5	7212
E14_5_1_1	E14.5	6529
E14_5_2_2	E14.5	6286
E16_5_1	E16.5	7259
E16_5_2_2	E16.5	7720
P 3_1_1	P3	4400
P 3_2_2	P3	5469

**Table 2:** Quality statistics of cell samples.

	E8.5	E10.5	E12.5	E14.5	E16.5	P3
**Viability (%)**	98	85	90	83	83	89
**Aggregation rate (%)**	<5	<5	<5	<5	<5	<5

### Single-cell transcriptomic analysis using the 10× Genomics Chromium

Single cells were prepared following 10 × Genomics, Inc (Pleasanton, CA) protocol. The protoplast suspension was loaded into Chromium microfluidic chips with 30 (v3) chemistry and barcoded with a 10 × Chromium Controller (10 × Genomics). According to the manufacturer’s instructions, RNA from the barcoded cells was subsequently reverse-transcribed, and sequencing libraries were constructed with reagents from a Chromium Single Cell 30 v3 reagent kit (10 × Genomics). Sequencing was performed with Illumina NovaSeq 6000 according to the manufacturer’s instructions (Illumina). FastQC (http://www.bioinformatics.babraham.ac.uk/projects/fastqc/) was used to perform basic statistics on the quality of the raw reads.

Raw reads were demultiplexed and mapped to the reference genome by the 10 × Genomics Cell Ranger pipeline (https://support.10xgenomics.com/single-cell-gene-expression/software/pipelines/latest/installation) using default parameters. Unless explicitly mentioned, all downstream single-cell analyses were performed using Cell Ranger and Seurat version 3.1.1 (https://remotes.r-lib.org/reference/install_version.html. In brief, unique molecule identifiers were counted for each gene and each cell barcode (filtered by Cell Ranger) to construct digital expression matrices^19^. In detail, cellranger count takes FASTQ files and performs alignment, filtering, barcode counting, and Unique Molecular Identifier (UMI) counting. It uses the Chromium cellular barcodes to generate feature barcode matrices.

### Initial cell typing in single-cell RNA sequencing data

To ensure robust and reliable transcriptomic signal-to-noise ratios without impairing sensitivity to small signals, we filtered out all cells with unique RNA counts (nUMI) <300, or distinct genes (nFeatures) <270 to remove under-sampled cells and simple cells such as erythrocytes. The ratio of mitochondrial transcripts to nuclear genome-derived transcripts is often used as a metric of cell stress or quality in scRNA-seq. 5% is typically used as a ceiling, but this is not supported across cell types and can fail to identify damaged cells, particularly cardiomyocytes, which can reach ~30%^20^ and exclude particular cardiomyocyte populations^21^. Given the changing nature of mitochondrial biogenesis across the embryonic to postnatal mouse heart^22^, we utilized a dynamically changing filter for mitochondrial transcript ratios: E8.5: 5%, E10.5: 5%, E12.5: 7.5%, E14.5: 10%, E16.5: 15%, P3: 20%. We normalized cell libraries through SCTransform, which accounts for the preservation of differential variation between highly variant and lowly variant genes^23^. We utilized Uniform Manifold Approximation and Projection (UMAP), following principal component analysis (PCA) and PCA dimension selection, to enable human-interpretable visualization of the transcriptomic space through dimensionality reduction. We visually examined the data for differences between batches of cells collected at each stage. Only P3 had significant batch differences. There are extensive suggested solutions for ‘correcting’ batch differences^24^. However, from a statistical fundamentals perspective, both a priori and empirically, such methods have been shown to produce aberrant downstream results^25^. We visualized UMAP in 3D,used Louvain clustering, and labelled clusters based on expression profiles. Further sub-clustering was performed as necessary, and a small number of cells were manually assigned where appropriate. The above was done using Seurat functions unless specified^26^.

## Data Records

The sequencing data from this study have been uploaded to the National Center for Biotechnology Information (NCBI) Sequence Reads Archive (SRA) with accession ID: PRJNA890252^27^. This includes 148 raw.fastq files for E8.5, E10.5, E12.5, E14.5, E16.5, and P3 stages. Matrix files on exonic and intronic expression can be accessed through the project accession number GSE230531 at the NCBI Gene Expression Omnibus^28^.

## Technical Validation

**Fig. 2:**
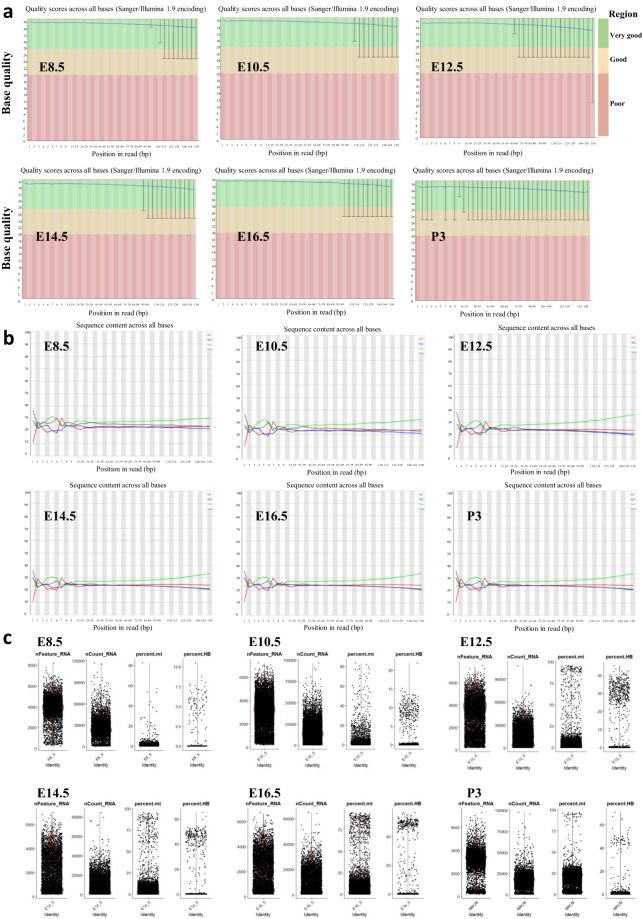
Quality control (QC) of single-cell data. (**a**) Raw data of base mass distribution map in E8.5, E10.5, E12.5, E14.5, E16.5, and P3. (**b**) Raw data of base content distribution map in E8.5, E10.5, E12.5, E14.5, E16.5, and P3. (**c**) Violin diagram illustrating the number of genes (nfeature_RNA), unique molecular identifier (UMI) (nCount_RNA), and the percentage of mitochondrial UMI (percent. mito) and erythrocyte UMI (percent. HB) in E8.5, E10.5, E12.5, E14.5, E16.5, and P3.

To validate the quality of the cDNA synthesis and barcoding steps, especially the DNA contamination, we first assessed the mapping location of aligned reads. As expected, the base quality of all stages is distributed in the green (very good) and yellow (good) areas, so it is considered that the base quality of the original data of all samples is good, and the data is within the applicable range (Figure 2a). Figure 2b shows the base content distribution diagram of sample read2 at E8.5, E10.5, E12.5, E14.5, E16.5 and P3, respectively. The abscissa represents the position of the base, and the ordinate represents the percentage of the base content. Green, red, blue and black correspond to bases A, T, C and G, respectively. Reads2 is stable throughout the sequencing process, and there is no significant AT or GC separation. Therefore, it can be considered that the base content distribution of all samples is normal.

In high-throughput sequencing, each base will have a corresponding quality value to measure the sequencing accuracy. The error rate of base quality value 30 is 0.1%. Q30 represents the percentage of bases with a quality value greater than or equal to 30. The higher Q30, the more accurate the sequencing. Table 3 shows that the Q30 of barcode, RNA and UMI sequences of almost all samples is greater than 90%, and the effective barcode accounts for a high proportion, which indicates that the sequencing data quality is high and can be used for subsequent analysis.

**Table 3:** Barcode, RNA, UMI sequence quality control.

	E8.5	E10.5	E12.5	E14.5	E16.5	P3
**Valid Barcodes (%)**	98.20	97.70	97.80	97.90	97.90	98.00
**Q30 Bases in Barcode (%)**	95.80	95.10	95.50	95.30	95.20	93.40
**Q30 Bases in RNA Read (%)**	91.50	90.50	90.60	91.00	90.70	86.70
**Q30 Bases in UMI (%)**	95.40	94.80	94.90	94.90	94.80	92.60

Table 4 shows that the percentage content of the reference genome aligned to the exonic region is the highest, and the content aligned to the intronic region or the intergenic region is shallow, which indicates that the obtained sequencing data is from RNA.

**Table 4:** Reference genome alignment.

	E8.5	E10.5	E12.5	E14.5	E16.5	P3
**Reads Mapped Confidently to Intergenic Regions (%)**	2.80	3.40	3.60	3.40	3.10	2.90
**Reads Mapped Confidently to Intronic Regions (%)**	7.30	8.70	6.80	11.00	8.20	7.30
**Reads Mapped Confidently to Exonic Regions (%)**	79.80	77.60	78.30	75.30	78.00	79.10
**Reads Mapped Confidently to Transcriptome (%)**	76.50	74.10	75.10	72.20	74.70	76.20
**Reads Mapped Antisense to Gene (%)**	0.90	1.00	0.60	0.50	0.50	0.60

Figure 2c shows the distribution of gene number (nfeature_RNA), UMI number (nCount_RNA), mitochondrial UMI proportion (percent. mito) and erythrocyte UMI proportion (percent. HB) of samples at six-time points by violin chart.

**Fig. 3:**
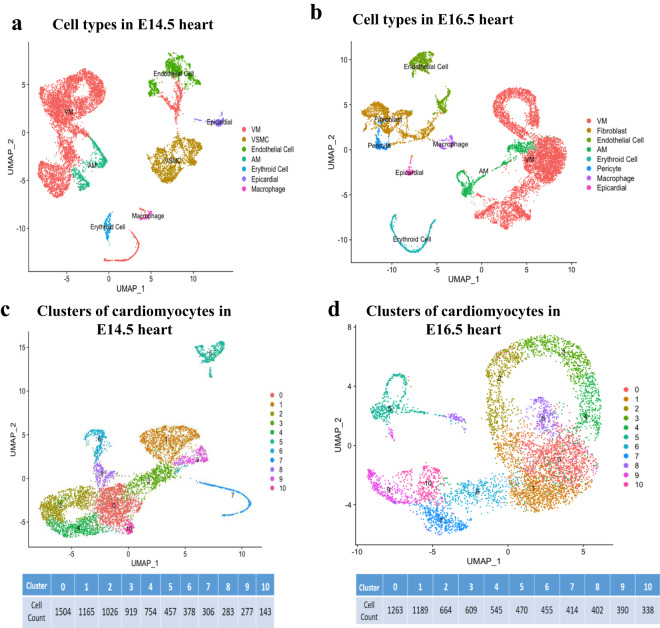
Subpopulations of cardiomyocytes and the developmental transcriptome of the CCS. (**a,b**) UMAP cell clustering results of cell types in E14.5 and E16.5 hearts. (**c,d**) Cardiomyocyte subpopulations of heart in E14.5 and E16.5 development stages.

After clustering cells by graph-based clustering method and visualizing by t-SNE dimension reduction, cell clustering results of the mouse embryo development process were obtained, and the number and type of cells in the subpopulation were displayed. Here, we take the sequencing results of E14.5 and E16.5 hearts as examples to analyse the transcriptional spectrum of CCS. As shown in Figure 3a,b, each dot represents a cell, and the color is used to distinguish different cell subpopulations. The closer the cell’s distance, the closer the gene expression profile is. Table 5 shows marker genes that define cardiomyocytes, fibroblasts, endothelial cells, macrophages, vascular smooth muscle cells (VSMC), epithelial cells, and mesoderm-derived cells. We found that the main cell types in the E14.4 heart include ventricular myocyte (VM), VSMC, endothelial cell, atrial myocyte (AM), erythroid cell, epicardial cell, macrophage, etc. But in addition to these cell types, the E16.5 heart also contains fibroblast and pericyte.

**Table 5:** Cell type assignment based on the marker genes reported in previous studies.

Cell type	Marker genes
Atrial myocyte	*Nppa*^13^, *Gja5*^29^
Ventricular myocyte	*Actn2*^30^, *Myh6*^13^, *Tnnt2*^13^
Cardiomyocyte	*Acta1*^32^, *Actc1*^33^*, Tnnc1*^32^, *Tnnc3*^32^
Cardiac conduction system	*Cacna2d2*^31^, *Hcn4*^31^, *Tbx3*^31^, *cacna1g*^31^, and *Ephb3*^31^
Endothelial cell	*Egfl7*^32^, *Epas*^32^*, Fabp4*^32^*, Flt1*^32^, *Pecam*^32^, *Tie1*^32^
Macrophage	*CD68*^32^, *CD74*^32^*, Lgals3*^32^*, Itgam*^32^
Smooth muscle cell	*Rgs5*^34^, *Tpm2*^35^
Erythroid cell	*Gata1*^36^, *Klf1*^36^, *Runx1*^37^, *Tal1*^37^, *Cldn6*^34^, *Cldn7*^34^, *Epcam*^38^
Mesoderm derived cell	*Cdh11*^39^, *Col3a1*^39^*, Pcolce*^39^

At the same time, the UMAP unsupervised dimensionality reduction and clustering algorithm was used to unbiased classify cardiomyocytes obtained from the classification of cardiac cells (Figure 3c,d). According to the number in the figure, we can see a total of 11 cardiomyocyte subgroups in both E14.5 and E16.5 hearts.

**Fig. 4:**
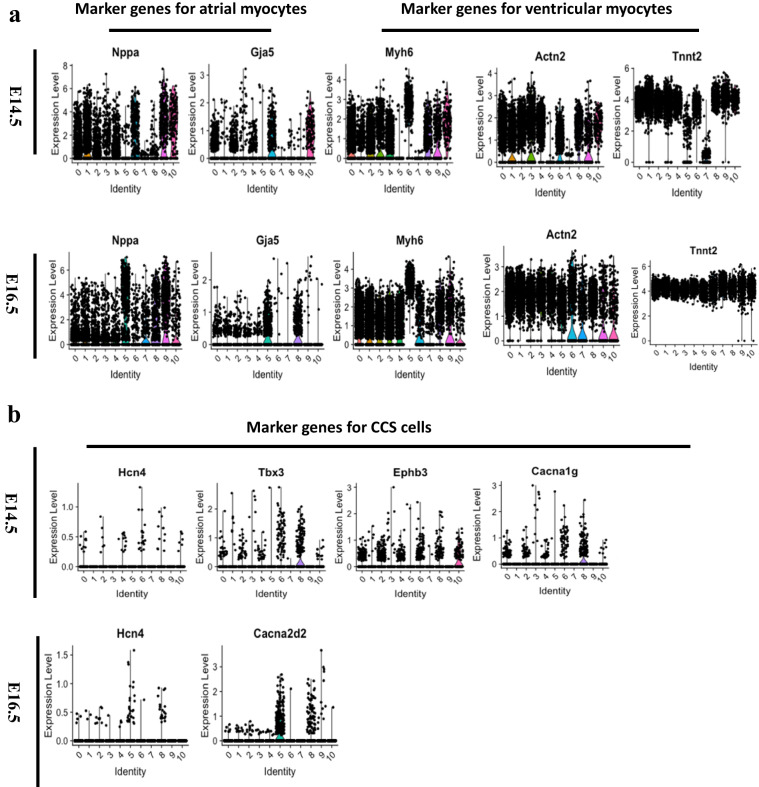
The distribution of marker genes for the atrial myocytes, ventricular myocytes and CCS cells at E14.5 and E16.5 development stages. (**a**) The marker genes of atrial myocytes (*Nppa* and *Gja5*) and ventricular myocytes (*Myh6*, *Actn2*, and *Tnnt2*) enriched in different clusters. (**b**) The marker genes of CCS cells (*Hcn4*, *Tbx3*, *cacna1g*, *Ephb3*, and *Cacna2d2*) expressed in different clusters. AM: atrial myocytes; VM: ventricular myocytes; CCS: cardiac conduction system.

Violin plots show that cell markers of atrial myocytes (*Nppa*, *Gja5*)^13,29^ (Figure 4a), ventricular myocytes (*Myh6*, *Actn2*, and *Tnnt2*)^13,30^ (Figure 4b) and cardiac conduction cells (*Hcn4*, *Tbx3*, *cacna1g*, *Ephb3*, and *Cacna2d2*)^31^ (Figure 4c) enriched in clusters.

## Data Availability

All single-cell RNA-Seq analyses were performed using FastQC (http://www.bioinformatics.babraham.ac.uk/projects/fastqc/), Cell Ranger (download from 10x genomics) and Seurat (https://satijalab.org/seurat/).
